# Microcrater-Arrayed Chemiluminescence Cell Chip to Boost Anti-Cancer Drug Administration in Zebrafish Tumor Xenograft Model

**DOI:** 10.3390/biology11010004

**Published:** 2021-12-21

**Authors:** Ching-Te Kuo, Yu-Sheng Lai, Siang-Rong Lu, Hsinyu Lee, Hsiu-Hao Chang

**Affiliations:** 1 Department of Mechanical and Electro-Mechanical Engineering, National Sun Yat-sen University, Kaohsiung 80424, Taiwan; 2 Department of Life Science, National Taiwan University, Taipei 10617, Taiwan; r05b21026@ntu.edu.tw (Y.-S.L.); ysking326@hotmail.com (S.-R.L.); hsinyu@ntu.edu.tw (H.L.); 3 Department of Pediatrics, National Taiwan University Hospital, National Taiwan University College of Medicine, Taipei 10617, Taiwan

**Keywords:** drug screening, zebrafish xenograft, microarray chip, chemiluminescence

## Abstract

**Simple Summary:**

Personalized drug cocktails have been widely implemented in cancer treatment, due to their merits of using a drug synergistic combination rather than a single effector. Through the incorporation of a cell microarray chip, the usage of both cell amount and reagent can be optimized and effectively reduced in cost. Herein, we present a laser direct writing strategy to fabricate a microcrater-arrayed (µCA) chip incorporated with an automatic liquid handling platform. Each cell droplet with a critical volume of 200 nL containing 100 SK-N-DZ or MCF-7 cells was utilized. The drug synergy screening took less than 30 s for a total of 81 tests. The results show that the potent drug prediction of the µCA chip is more accurate than the conventional 96-well plate assay, which are all compared using zebrafish DiI-labelled tumor xenograft models. Taken together, these findings may impact high-throughput drug screening and personalized drug medicine.

**Abstract:**

Purpose: The aim of this study was to develop a rapid and automatic drug screening platform using microcrater-arrayed (µCA) cell chips. Methods: The µCA chip was fabricated using a laser direct writing technique. The fabrication time required for one 9 × 9 microarray wax chip was as quick as 1 min. On a nanodroplet handling platform, the chip was pre-coated with anti-cancer drugs, including cyclophosphamide, cisplatin, doxorubicin, oncovin, etoposide, and 5-fluorouracil, and their associated mixtures. Cell droplets containing 100 SK-N-DZ or MCF-7 cells were then loaded onto the chip. Cell viability was examined directly through a chemiluminescence assay on the chip using the CellTiter-Glo assay. Results: The time needed for the drug screening assay was demonstrated to be less than 30 s for a total of 81 tests. The prediction of optimal drug synergy from the µCA chip was found by matching it to that of the zebrafish MCF-7 tumor xenograft model, instead of the conventional 96-well plate assay. In addition, the critical reagent volume and cell number for each µCA chip test were 200 nL and 100 cells, respectively, which were significantly lower than 100 µL and 4000 cells, which were achieved using the 96-well assay. Conclusion: Our study for the µCA chip platform could improve the high-throughput drug synergy screening targeting the applications of tumor cell biology.

## 1. Introduction

Cell-based microarray chips have been widely utilized to explain the gaps in limited cell sources, critical reagent volumes, and high-throughput and high-content drug screening [1,2,3,4,5,6]. Based on these merits, cells cultured in microscales would possess drug responses resembling those in vivo microenvironments. This may be attributed to the higher cell-to-volume ratio than that of macroscale cell cultures [7,8]. Moreover, the study of cancer stem cells (CSCs) incorporated with cell microarrays has the potential to address the tumor heterogeneity [9]. 

The screening potential of optimal drug combinations for high-throughput and high-content drug screening has been widely studied. For example, the adoption of a feedback control system (FSC) could speed the screening up to 10,000 times more than conventional drug screening assays [10,11,12]. Large-scale cell simulations could contribute more efficacy to in vivo drug prediction than conventional approaches [13,14]. All of these approaches were accomplished using multi-well plates. The use of such plates would typically acquire a large number of cells, which may counter the feasibility of clinical medicine with limited biopsies [15]. Although the multilayered 3D cell culture has been revealed with a respective efficacy targeting tumor microenvironments, both the cell numbers and the testing period are still of concern [16]. 

Microfluidic technology has attracted numerous potential applications in anti-cancer drug screening, due to its compact size, extremely low dose use, and ready integration with high-throughput analysis setups [17,18]. For example, continuously perfusion-based microfluidic devices could provide a dynamic examination of shear stress to cells and a flow medium exchangeable condition [19,20]. However, the device channel layout and/or the exterior pump–valve system might become complicated if the reagents and their relevant combinations increase. An improved alternative approach to address the above issue could be droplet-based microfluidics [21,22]. This technique typically uses an oil-covered cell and/or drug-contained droplet array. The encapsulation rate of cells ranges from Hz (for multi-drug combination) to kHz (single-drug screening) [21,23]. Although such drug testing is highly efficient, it might complicate the post cell culture application, such as extracting the cells from the oil-covered substrate for further gene expression [24]. 

Prior to launching a new drug, mouse animal models have been widely adopted for the pre-clinical trials because of their biological relevance to humans, ability to mimic disease responses in patients, and the great potential for transgenic analysis compared with human trials [25]. Previously, we developed a nanodroplet processing platform incorporated with a mouse model for the evaluation of optimal drug combinations [26,27]. This demonstrated that drug evaluation is superior to using a conventional 96-well plate assay. Nonetheless, this mouse model presents several drawbacks, particularly for routine clinical assays. For example, the number of cells required for engrafting is typically high, reducing the capacity for high-throughput screening with drugs. In addition, the time required for drug evaluation and the expansion of colonies would last months, thus hindering its feasibility for clinical assays and personalized medicine. 

In recent decades, zebrafish xenografts have been demonstrated to be an attractive alternative to mice. The zebrafish model presents a rapid assay platform, suitable for cellular resolution and drug target identification, and the ability to perform a vast number of transplants [28,29,30]. In addition, this model can easily be used in high-throughput screening using an existing liquid-handling machine. Moreover, the cost of maintaining such a species is critically low (1%) as compared to that needed to maintain mice [25]. 

To address the technology gaps described above, herein we present a microcrater-arrayed cell chip (µCA chip) for facilitating the high-throughput screening of optimal drug combinations. The chip was fabricated using a configurable laser directing writing on a molten wax membrane to perform the arrayed microcrater wells. The array chip was fabricated 60 times (1 min for one 9 × 9 array chip) faster than our previously developed nanodroplet platform (1 h for one array chip) [27]. We demonstrated that the µCA chip can present an in vivo efficacy more accurately as compared to that derived from conventional 96-well plates, which was validated using a zebrafish model. Altogether, these findings highlight that the µCA chip platform could be a cost-effective and high-throughput toolkit for streamlining pre-clinical drug screening efficacy. 

## 2. Materials and Methods

### 2.1. Coating with Waxy Film on Glass Substrate

The procedures for coating the waxy film on the substrate were partially modified from our previously developed *ParaStamp* technique [26]. First, while uniformly pressuring the sandwiched set of a foil sheet, a thermoresponsive sheet (Parafilm “M”) and a plain PDMS sheet (10:1 *w/w* base to cure, SYLGARD 184; Dow Corning, Midland, MI, USA) was performed at 70 °C for 30 s. Afterwards, the PDMS sheet absorbed with liquid-phase paraffin wax was peeled off and immediately stamped onto the examined substrates for another 30 s at room temperature. A glass slide (511614, Muto Pure Chemical Co., Ltd., Tokyo, Japan) was used as the substrate. The waxy film coated onto substrates with a thickness of approximately 1 µm, after peeling off the PDMS stamper. 

### 2.2. Laser Direct Writing on Waxy Film-Coated Slide Substrate

The activity of laser writing on the examined substrates was performed using a CO_2_ laser engraver (Nova-35, Thunderlaser; 30 W, Guangdong, China). The maximum energy density of the laser was stated to be 3250 J/mm^2^. Parameters adopted for patterning circular shapes were set as “drill hole” modes, and the drilling time was set from 0.0005 s to 0.3 s with 4% power and a 10 mm/s moving velocity. The corresponding energy densities applied could be evaluated as 0.065 to 39 J/mm^2^. For patterning with line shapes, parameters were set as “cutting” modes, and the moving velocities were set from 5 mm/s to 70 mm/s with 4% power and an identical length of 10 mm. The energy densities were then evaluated as 260 to 18.6 J/mm^2^ accordingly. 

### 2.3. Microcrater-Arrayed (µCA) Chip

A 9 × 9 array with circular patterns was first performed on a waxy film-coated glass slide by using the laser writing technique described above. The parameter was set as “drill hole” mode, and the drilling time was set as 0.2 s with a 4% laser power and a 10 mm/s moving velocity. This resulted in an energy density of 26 J/mm^2^, and the measured diameter of the patterned circles was approximately 900 µm. The center-to-center distance between nearby patterns was 2.2 mm. Neuroblastoma SK-N-DZ (CRL-2149, ATCC) and breast cancer MCF-7 (HTB-22, ATCC) cells were used for the proof-of-concept demonstration with the anti-cancer drugs cyclophosphamide (Cyp; Baxter Oncology, Halle, Germany), cisplatin (Cis; Fresenius Kabi, Solan, Himachal Pradesh, India), doxorubicin (Dox; Adriamycin, Pfizer, New York, NY, USA), oncovin (Onc), etoposide (Eto; Fresenius Kabi, Solan, Himachal Pradesh, India), and 5-fluorouracil (5-Fu; Haupt Pharma, Wolfratshausen, Germany). SK-N-DZ cells were maintained in Dulbecco’s modified Eagle medium (DMEM; SH30022.02, HyClone, Taipei, Taiwan), supplemented with 10% FBS and 1% P/S. MCF-7 cells were maintained in α-minimum essential medium (α-MEM; SH30265, GE Healthcare), supplemented with 10% FBS and 1% P/S. 

For drug screening, first, all drug droplets with designed concentrations were dispensed onto the corresponding wells carried by a programmable liquid dispenser (Versa 10 spotter, Aurora Instrument Ltd., Vancouver, Canada). Drugs were initially diluted in deionized (DI) water, and the volume of each droplet was set to 200 nL. The chip was then placed at room temperature for 10 min to dehydrate the drug droplets. Subsequently, cell droplets with a nL volume of 200 (100 cells per droplet) were dispensed into the well and then incubated for 1 day. Live cells were assessed by staining with CellTiter-Glo (G7570, Promega, Wisconsin, USA) and detected using a luminescent image analyzer (TCX-LS13, FluorChemM, NTU, Taipei, Taiwan; exposure time: 30 s). Cell viability with corresponding luminescent intensity was evaluated using a Fiji imaging macro software and normalized to the untreated cells. The IC_50_ values were fitted from a four-parameter logistic equation:

(1) $$Y=Y_{min}+\frac{Y_{max}-Y_{min}}{\left(1+{\left(\frac{IC_{50}}{X}\right)}^{HillSlope}\right)}$$

where *Y* is the normalized cell viability; *X* is the drug concentration; and *Y_max_* and *Y_min_* are the upper and lower plateaus of the curve of dose–response, respectively. *Y_max_* was set as the control output, and *Y_min_* was set as 0. *HillSlope* defined the steepness of a drug response profile. 

For drug evaluation performed in standard 96-well plates, media containing cancer cells with a density of 4000 cells per well were tested. The concentrated drug solutions were then added to the corresponding wells, leading to the equivalent drug concentrations compared to those used in the cell microarray assay. The final volume was 100 µL per well. A plate reader was used to determine the cell viability following the MTT (3-[4,5-dimethylthiazol-2-y1]-2,5-diphenyl tetrazolium bromide;) assay (Abcam, Cambridge, UK). 

### 2.4. Zebrafish Xenograft Model

Zebrafish (wild-type Danio rerio) were handled (temperature, 28 °C; pH 7.2–7.4; 14 h on and 10 h off light cycle) according to standard protocols as well as NTU regulations of the Animal Care and Use Committee. Zebrafish embryos were obtained from mating adults under standard mating conditions. Prior to transplantation with MCF-7 cells (300 cells in 0.01 µL of cell culture medium), 48 h post fertilization (hpf) zebrafish embryos were anesthetized (with 0.04 mg/mL tricaine; Sigma, Taipei, Taiwan) and then transferred to a 0.1% agarose-coated dish for cell injection. DiI-labeled cells in 10 nL of cell suspension were injected into the yolk sac of the anesthetized embryos at room temperature, after which the xenografts were kept at 34 °C until the end of experiment. Cell tracker DiI dye (Thermo Fisher, Waltham, MA, USA) is a fluorescent dye (excitation at 553 nm and emission at 570 nm) available for monitoring cell movement or location during long-term observation and was well suited for the zebrafish models. Injected embryos were transferred to a 96-well plate (one embryo/well) containing drug diluted in 200 μL of E3 media (without methylene blue) 1-day post injection (dpi). Following this, the zebrafish xenografts were randomly distributed into the drug treatment groups designed for 2 days. The detection of fluorescence intensity (corresponding to the tumor growth rate) was performed at 1 dpi and 3 dpi using a fluorescence microscope using identical acquisition settings. The images were analyzed using Fiji ImageJ software. At the end of the experiments, embryos were euthanized by tricaine overdose. 

### 2.5. Zebrafish Embryo Toxicity (ZFET) Testing 

Acute toxicity testing on zebrafish embryos was performed following the Organization for Economic Co-operation and Development (OECD) guidelines for the fish embryo toxicity (FET) test on embryos. We used 48 h post fecundation (hpf) zebrafish embryos to perform the test. The embryos were treated with the designed drugs for 96 h. The tested concentrations were as follows: 10 (33.2 μM), 20 (66.4 μM), 30 (99.6 μM), 50 (166 μM), and 100 (332 μM) μg/mL for Cis; 1000 (7760 μM), 2000 (15,520 μM), 3000 (23,280 μM), 5000 (38,800 μM), and 10000 (77,600 μM) μg/mL for 5-Fu; 100 (383 μM), 200 (766 μM), 400 (1532 μM), 500 (1915 μM), and 1000 (3830 μM) μg/mL for Cyp; 5 (8.5 μM), 10 (17 μM), 20 (34 μM), 50 (85 μM), and 100 (170 μM) μg/mL for Eto. The embryos were examined to determine mortality and the maximum non-lethal concentration (MNLC). Each test was performed for 20 embryos. The MNLC for fish was defined as the mortality rate ≤10%. 

### 2.6. Statistical Analysis 

Student’s *t* test was used to compare data from the two groups. Tukey HSD and one-way ANOVA tests were used to compare data from more than two groups. *p* < 0.05 was considered to be statistically significant.

## 3. Results and Discussion

### 3.1. Laser Direct Patterning of Microcrater Wax Structures

**Figure 1:**
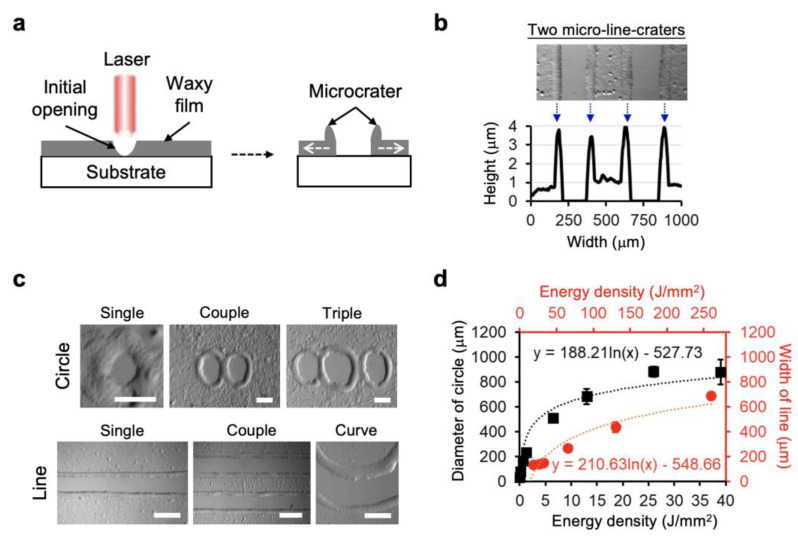
Laser direct writing on waxy films coated on glass slide substrates: (**a**) Illustration showing the waxy microcrater patterns carried by laser direct writing. (**b**) Surface profile of the two typical micro-line craters by laser writing on the waxy film-coated substrate. (**c**) Representative micrographs showing the capability of fabricating both circle and line patterns on the substrate by laser writing. Scale bars are 50 µm in top panel and 200 µm in bottom panel. (**d**) Relationship between the fabricated size of circle (black color) or line (red color) pattern and the energy density applied by the CO_2_ laser. Data represent the mean ± SD, *n* = 3 for circle pattern and *n* = 4 for line pattern.

We employed a CO_2_ laser to realize laser direct writing on wax-coated substrates. A schematic of the working principle is shown in Figure 1a. Figure 1b,c demonstrate the characteristics of laser direct patterning on the wax-coated glass substrate upon applying 0.13 J/mm^2^ of dot laser energy. With sufficient laser energy applied, an opening in the waxy film was achieved from the glass slide substrate. The fabricated patterns typically possessed a crater-like microstructure along with the boundary, with a height of 4 µm. This suggests that the microcrater resulted from the accumulation of molten wax around the boundary. A dimensional saturation of fabricating the circle or line pattern is shown in Figure 1d, indicating that the transfer distance of heat induced by the laser could not sustain the patterning with a larger size. 

**Figure 2:**
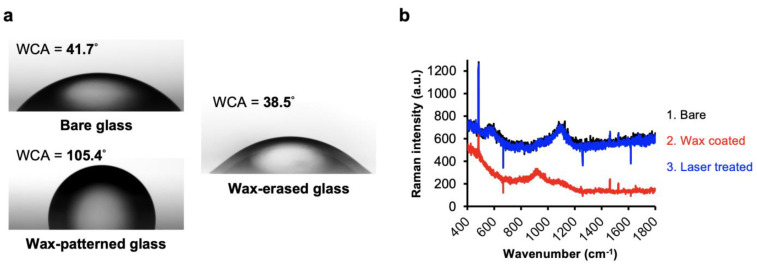
Complete removal of the patterned wax films by laser direct writing: (**a**) Comparison of water contact angle (WCA) measured from bare glass, wax-patterned glass, and wax-erased glass by CO_2_ laser. (**b**) Raman spectra of the glass slide substrate during the three different treatments, including bare substrate (black), wax coating (red), and laser writing (blue).

To further compare the patterning efficiency, that is, whether there is relatively less or no residual wax within the pattern, we compared the water contact angle (WCA) and the surface material composition using Raman spectra (iHR550 Micro-Raman spectrometer, HORIBA, Taiwan) with and without laser treatments (Figure 2a,b). The results show that the WCAs measured from glass substrates presented a significant difference between the wax-coated and the laser-treated surfaces. A significant removal of waxy film was detected, as shown in Figure 2b. 

### 3.2. High-Throughput Drug Screening by Microcrater-Arrayed (µCA) Chip

**Figure 3:**
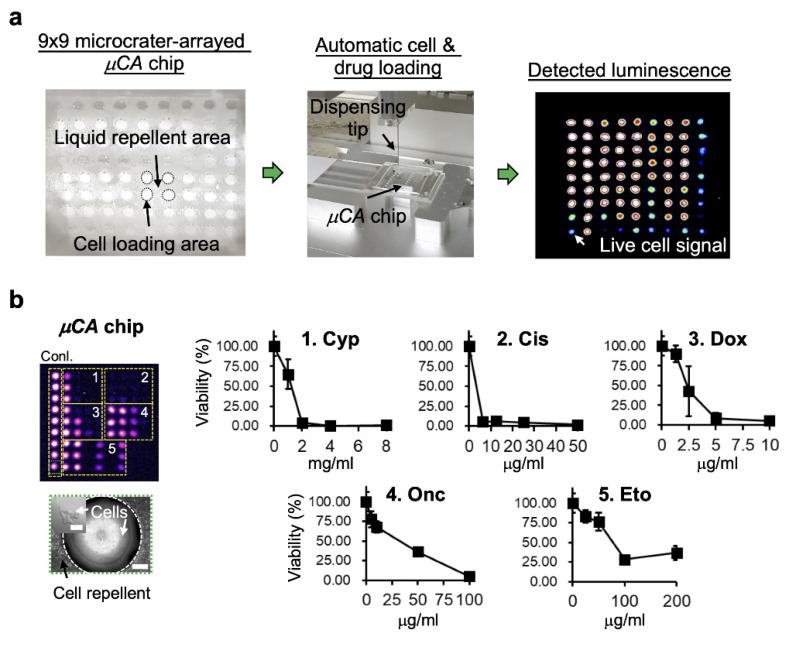
High-throughput drug screening by the microcrater-arrayed (µCA) chips: (**a**) Experimental procedure of the drug screening. (**b**) Demonstration for the high-throughput drug screening on SK-N-DZ cells by the µCA chip. Live cells were evaluated based on the detection of chemiluminescence. Cyp: cyclophosphamide; Cis: cisplatin; Dox: doxorubicin; Onc: oncovin; Eto: etoposide. Scale bar is 170 µm, and the insert bar is 50 µm. Data represent the mean ± SD, *n* = 3 independent experiments.

The µCA chip with microcrater well structures was fabricated by laser direct writing (Figure 1a). The details can be found in the experimental section. The chip was mounted on a programmable XYZ positioning stage and operated using an automatic microdroplet dispenser for high-throughput drug synergy screening. A PDMS glass gasket was piggybacked to prevent the evaporation of the microdroplets during the experiments. Cell viability was determined by staining the cells with chemiluminescence, and the time spent for such evaluation was less than one minute (Figure 3a). Figure 3b shows that the pre-tested SK-N-DZ cancer cells were grown on the µCA chip, behaving with proper cell morphology compared with those cultured in a 96-well plate (see also Figure S1). In addition, it presents that the µCA chip system successfully demonstrated potential for high-throughput drug screening with Cyp, Cis, Dox, Onc, and Eto, commonly used anti-cancer drugs. 

**Figure 4:**
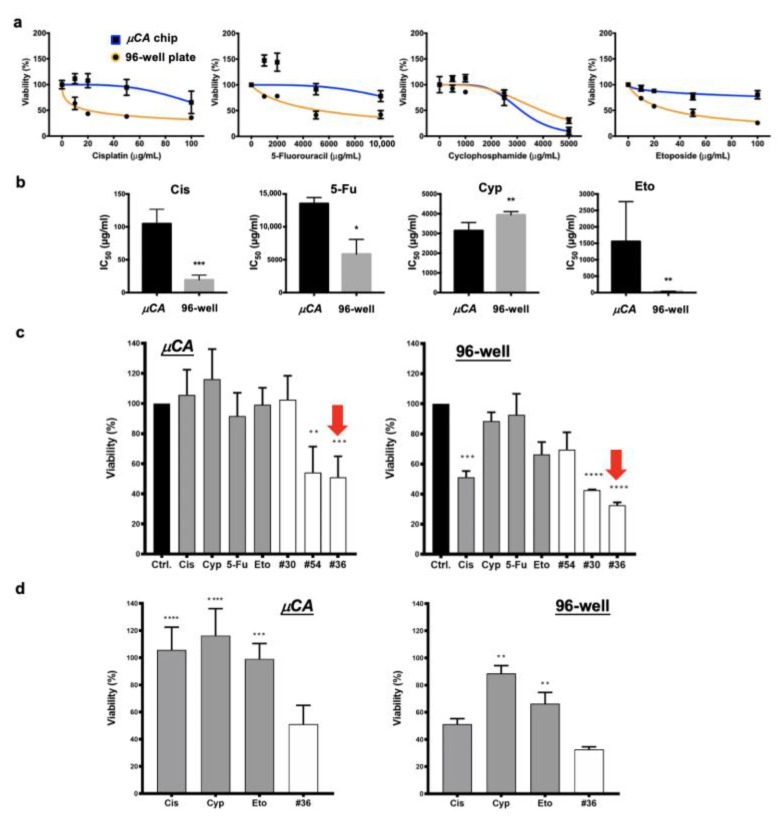
Comparisons of drug testing derived from the µCA chip and the conventional 96-well plate: (**a**) Drug profiles of 24 h cisplatin (Cis), 5-fluorouracil (5-Fu), cyclophosphamide (Cyp), and etoposide (Eto) acting on MCF-7 cells, which are derived from µCA chips and 96-well plates, respectively. The lowest drug concentration used for each screening was 0 µg/mL. (**b**) Comparison of the drug IC_50_ values derived from µCA chip and 96-well plate results (Figure 4a). (**c**) Comparison of cell viabilities obtained from the optimal drug combinations and the corresponding single-drug treatments. The single-drug concentrations were 15 µg/mL (49.8 μM), 400 µg/mL (1532 μM), 3000 µg/mL (23,280 μM), and 10 µg/mL (17 μM) for Cis, Cyp, 5-Fu, and Eto, respectively. All data were compared to the control. The optima were selected from #30, #36, and #54 groups; please refer Supplementary Figures S2 and S3 for more detail. The red arrows represent the optimized drug combinations. (**d**) Comparisons of cell viabilities obtained from the optimized #36 combination and the corresponding single-drug treatments. Data represent the mean ±SD, *n* = 3 independent experiments. * *p* < 0.05, ** *p* < 0.01, *** *p* < 0.001, and **** *p* < 0.0001.

Figure 4 shows that the comparison between drug synergy screening for another cancer cell line (MCF-7) and the µCA chip and the conventional 96-well plate assay. After Cis, 5-Fu, Cyp, and Eto, the four anti-cancer drugs, were treated, the distinct toxicity profiles found among them are present in Figure 4a. The drug IC_50_ values evaluated from Figure 4a were also distinct (Figure 4b). The IC_50_ values derived from the chip were 121.6 ± 34.9 µg/mL (403.7 ± 115.9 μM), 13,767 ± 775 µg/mL (106,832 ± 6014 μM), 3195 ± 381 µg/mL (12,237 ± 1459 μM), and 1592 ± 1209 µg/mL (2706 ± 2055 μM) for Cis, 5-Fu, Cyp, and Eto, respectively. The IC_50_ values derived from the 96-well plate were 20.3 ± 3.8 µg/mL (67.4 ± 12.6 μM), 5950 ± 2042 µg/mL (46,172 ± 15,846 μM), 3928 ± 240 µg/mL (15,044 ± 919 μM), and 33.0 ± 2.0 µg/mL (56.1 ± 3.4 μM) for Cis, 5-Fu, Cyp, and Eto, respectively. From the 96-well plate assay, the IC_50_ values of Cis, 5-Fu and Eto were significantly lower than those of the µCA chip. Instead, the Cyp-treated cells derived a higher IC_50_ value from the 96-well plate assay than the µCA chip. Figure 4c compares the cell viabilities that were derived from the µCA chip and the 96-well plate. These comparisons were based on the three representative drug treatments with high, moderate, and low inhibiting efficiency, which were selected from Figures S2 and S3, for the µCA chip (#36, #54, and #30) and 96-well plate (#36, #30, and #54), respectively. The single-drug treatments corresponded to the selected drug combinations. Drug combination #36 (15 µg/mL of Cis, 400 µg/mL of Cyp, and 10 µg/mL of Eto) achieved the highest inhibiting rate on MCF-7 cells from both the µCA chip and the 96-well plate. Noteworthily, the other two drug combinations of #30 (15 µg/mL of Cis, and 10 µg/mL of Eto) and #54 (15 µg/mL of Cis, 3000 µg/mL of 5-Fu, 400 µg/mL of Cyp, and 10 µg/mL of Eto), derived from the two screening platforms, contradicted with each other. Drug combination #54 contributed to a moderate cell inhibiting rate in the µCA chip (** *p* < 0.01), whereas no significant inhibition was achieved by such a drug combination in the 96-well plate. 

### 3.3. Successful Translation of the Selected Optimal Drug Combination into Zebrafish Tumor Xenograft Model 

**Figure 5:**
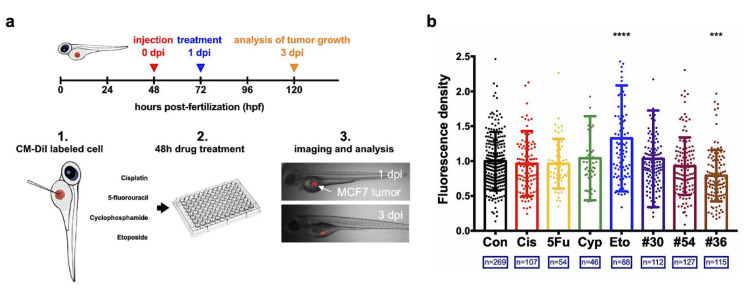
Verification of the drug combination efficacy via the in vivo zebrafish tumor xenograft models: (**a**) Procedure of the zebrafish xenograft assay; MCF-7 cells were used for this model. (**b**) In vivo tumor inhibiting rates of the evaluated drug combinations from the µCA chip and the corresponding single drugs used. The concentrations of cisplatin (Cis), 5-fluorouracil (5Fu), cyclophosphamide (Cyp), and etoposide (Eto) were 15 µg/mL (49.8 μM), 3000 µg/mL (23,280 μM), 400 (1532 μM) µg/mL, and 10 µg/mL (17 μM), respectively. Groups #30, #36 and #54 are given in Figures S2 and S3. “n” indicates the total number of zebrafishes used for each assay. Each datum was normalized by the average fluorescence density (intensity/pixel2) of control group (Con). *** *p* < 0.001 and **** *p* < 0.0001.

To leverage the efficiency of comparing in vitro and in vivo assays, zebrafish tumor xenograft models were used for comparison (Figure 5a). Engrafted MCF-7 tumors with DiI labeling were easily detected by fluorescence microscopy and exactly matched the location of tumors observed in bright-field images. The results show that the screened optimal drug combination #36 from the µCA chip presented significant tumor inhibition, as compared with the control (Figure 5b). Nonetheless, no significant difference was observed among the single drugs administrated, except that the drug Eto likely improved the tumor growth. In addition, drug combination #54 attenuated the in vivo tumor growth, although the inhibition rate was not significantly different from that of the control. Instead, there was no significant attenuation of tumor growth when drug combination #30 was adopted. The detailed characteristics of the three assays are presented in Table 1. 

It is well known that the drug uptake in zebrafish models may differ from that of mammals, especially through the gill cell barrier and/or intestine. Despite the above issue, many compounds have been revealed to attenuate disease in a similar way to mouse models, such as 5-Fu [30]. In this paper, we adopted the zebrafish model to compare the drug predictions from the well plate and microchip assays due to advantages such as speed and the ability to perform a large number of transplants. For the demonstration comparing the drug synergy in our developed system, the selected single-drug concentration was mainly based on both the MNLC and the resulting viability, which was greater than that of the IC_50_. The MNLC for Cis, 5-Fu, Cyp, and Eto were 50 µg/mL (166 μM), 3000 µg/mL (23,280 μM), 400 µg/mL(1532 μM), and 20 µg/mL (34 μM), respectively. In future research comparing these results with human samples, we will correlate the selections with human plasma concentrations. 

The evaluation of cell viability using DiI labeling in zebrafish xenograft models has been widely used for different cancer cell models, such as melanoma cells [31] and leukemia cells [32]. Furthermore, this labeling can be applied for high-throughput drug screening by incorporating an automatic fluorescent microscopy system [33,34]. In this study, we adopted a similar approach to examine the in vivo cell viability. The changes in tumor area and fluorescent intensity were correlated with the cancer growth, which is in line with the results from other research groups [31,32,33,34]. 

**Figure 6:**
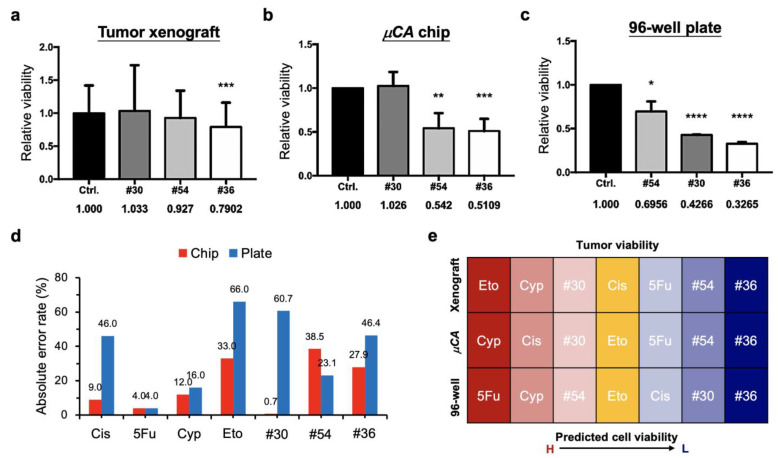
Efficacy of the optimal drug combinations inhibiting MCF-7 cells and tumors cultured in different models: (**a**) The zebrafish tumor xenograft model. (**b**) The µCA chip model. (**c**) The 96-well plate model. (**d**) Comparison of the absolute error rates derived from the µCA chip and the 96-well plate. The values are defined as the different rates of cell viability compared to those derived from xenografts. (**e**) Comparison of the tumor-inhibiting yield derived from xenograft, µCA chip, and 96-well plate. Drug combinations (#30, #36, and #54) and the corresponding single drugs used can be seen in Figure 5b. Results show that the in vitro drug efficacy (17.9% error rate and 57.1% prediction rate) derived from the µCA chip partially simulates the in vivo zebrafish results, compared to 96-well plate model (37.5% error rate and 28.6% prediction rate). Control groups are denoted as Ctrl. Numbers of zebrafish used for each test can be seen in Figure 5b. * *p* < 0.05, ** *p* < 0.01, *** *p* < 0.001, and **** *p* < 0.0001.

Figure 6 compares the tumor toxicity efficacy of the selected drug combinations for the zebrafish tumor xenograft model (Figure 6a), the µCA chip (Figure 6b), and the 96-well plate (Figure 6c) assay. Several results are noteworthy. First, drug combination #36 possessed the highest and most significant tumor inhibition rates among all the three assays. Second, no significant differences in tumor inhibition were observed between the zebrafish model and the µCA chip after administration of drug combination #30. In contrast, significant tumor inhibition by the same drug combination was observed in the 96-well plate assay. Third, drug combination #54 contributed to significant tumor inhibition in both the µCA chip and the 96-well plate. No effect of drug combination #54 was observed in the zebrafish model. 

CO_2_ laser micromachining has been widely adopted in numerous biomedical applications, owing to its rapid and maskless prototyping potential [35,36,37,38]. In this study, we successfully incorporated this technique with a thermoresponsive wax membrane to achieve applicable patterns on glass substrates (Figure 1). The particularly geometric features of the crater-like patterns mainly depend on the surface properties of the glass substrates.

Our previous study demonstrated that the microscale cell-culture microenvironment can benefit drug screening efficacy, as compared with a 96-well plate assay and an in vivo mouse model [27]. This revealed that the drug response using such a microenvironment resembles the in vivo mouse model. In this study, remarkedly, our new findings from using the µCA chip for drug synergy screening also present better agreement with data from in vivo zebrafish models, as compared to a 96-well plate assay (Figure 6d). The absolute error rate of predicting tumor viability presented an average error of 17.9%, lower than that of the well plate at 37.5%. In addition, it demonstrates that all the three selected drug combinations behave as predicated by screening from both the chip and the zebrafish assay, i.e., drug combinations #30, #54 # 36 present the lowest, the moderate, and the highest tumor inhibition (Figure 6e). In contrast, the prediction tendency of drug combinations #54 and #30 from the 96-well plate is contrary to that of the other screening platforms. The overall prediction yield of cell viability was 57.1% (four accurate tests out of seven) in our chip platform, which is better than (two accurate tests out of seven) that of the well plate, at 28.6% (Figure 6e). This indicates that the µCA chip is a better predictor of in vivo drug–tumor interactions than the conventional 96-well plate. In addition, the cell volume density of the 96-well plate was as low as 40 cells/µL, as compared with the µCA chip at 500 cells/µL and the zebrafish model at 30,000 cells/µL (Table 1). Although the cell surface densities of the µCA chip and the 96-well plate were likely equivalent, the µCA chip dominated the output of the drug synergy screening. Moreover, the critical volume of 200 nL per µCA chip test is significantly smaller than the recommended working volume of 1.5 µL for the standard 1536-well plate. 

Three-dimensional (3D) cell cultures were demonstrated to be able to reflect the in vivo-like microenvironment more effectively [39]. Our previous data revealed that 3D MCF7 spheroids (cell volume density: 450 cells/µL) possess higher cell viability (~110%) compared to that of two-dimensional (2D) cell cultures under a 5-µg/mL paclitaxel treatment, which reached ~75% of [40]. Similarly, head and neck squamous OSC19 cells even possessed higher cell viability of ~130% compared to that of a 2D culture under the same drug treatment, which reached ~75% [40]. Moreover, 3D SK-N-DZ cell cultures under some specific combinational treatments of cisplatin and MG132 proteasome inhibitor would cause them to acquire higher cell viability (even up to ~150%) than control cells [41]. In this paper, we also observed a discrepancy in greater cell viability than the control cells, which is not evidently observed in 96-well plate cultures (Figures S2 and S3). The cell volume density used in our current chip platform was 500 cells/µL, which reflects the similar density of 450 cells/µL used for previous 3D cell cultures (Table 1). For the 96-well plate, instead, a relatively smaller density of 40 cells/µL was used. We might suggest that the chip platform proposed in this paper could partially mimic the 3D cell environment, mainly based on the similar drug-to-cell volume density. 

In this paper, the tested drugs were first dispensed onto the surfaces of microarrays and then dried, which was followed by the loading of cell suspensions. This might result in the distinction of drug impact on cells as compared with the 96-well plate assay. The re-dissolved drugs might cause lower concentrations acting on cells, even though we previously revealed that the “first-deposition” and “post-deposition” of Cis drugs in 96-well plates have no significant difference in their drug response [27]. Many drugs from high-throughput screening (HTS) libraries have low solubility in media and are typically dissolved in DMSO, which is not easy to evaporate during the first deposition of the relevant drugs. It might further cause toxicity to cells. In future research, if patient biopsies are used, it would be more valuable to test the combinational therapy of water-soluble drugs in our system. 

The other reason causing the above distinction might be due to using different assay kits, including MTT for 96-well plates, CellTiter-Glo for microchips, and CM-DiI for zebrafish xenografts. Considering all the above discussions, indeed, the chip platform’s prediction of drug treatment partially reflects the xenograft results better than the multi-well systems. 

Most importantly, the time required for chip fabrication by laser direct writing in this study was 60 times faster than our previous approach using wax stamping (1 min versus 60 min, respectively) [27]. In addition, the time for evaluating 81 cell viabilities by chemiluminescence assay on the chip was as quick as 30 s, whereas our previous approach required 3 to 4 h for imaging and counting live-cell fluorescence. 

## 4. Conclusions

We have presented a microcrater-arrayed cell chip (µCA chip) to improve anti-cancer drug synergy screening. In addition, this approach was successfully demonstrated using a zebrafish tumor xenograft model. All of these outputs were provided by the developed µCA chip platform. The results show that the potent drug prediction of the µCA was more accurate than the conventional 96-well plate assay, which was compared with the zebrafish tumor xenograft models. In conclusion, our study opens a new avenue for high-throughput drug synergy screening, which is anticipated to ignite innovative applications for tumor cell biology. 

## Figures and Tables

**Table 1:** Characteristics of the µCA chip, 96-well plate, and zebrafish model.

Characteristics		µCA Chip	96-Well Plate	Zebrafish Model
	Cell number	100	4000	300
Culture scale (per well)	Volume of medium	0.2 µL	100 µL	0.01 µL
	Volume density	500 cells µL^−1^	40 cells µL^−1^	30,000 cells µL^−1^
	Surface area	6.4 × 10^5^ µm^2^	3.2 × 10^7^ µm^2^	Nil
	Surface density	1.6 × 10^−4^ cells µm^−2^	1.3 × 10^−4^ cells µm^−2^	Nil
Material	Substrate	Glass	Polystyrene	Nil
	Well	Wax	Polystyrene	Nil
	Total product area	484 mm^2^ (for 9 × 9 wells)	10,920 mm^2^ (for 12 × 8 well)	Nil
	Viability assay	Cell-Titer Glo	MTT assay	DiI

## Data Availability

The data presented in this paper are available on request from the corresponding authors.

## Supplementary Materials

The following are available online at https://www.mdpi.com/article/10.3390/biology11010004/s1, Figure S1: Cell morphology derived from the μCA chip and 96-well plate, Figure S2: Cell viability of MCF-7 cells during the cocktail treatments by the µCA chip, Figure S3: Cell viability of MCF-7 cells under cocktail treatments by the 96-well plate.
