# Supplementary material for: Microcrater-Arrayed Chemiluminescence Cell Chip to Boost Anti-Cancer Drug Administration in Zebrafish Tumor Xenograft Model
Source: Biology (Basel). 2021 Dec 21;11(1):4. doi: 10.3390/biology11010004 (PMC8773422; doi:10.3390/biology11010004)
Supplement: Supplementary file 1 [file biology-11-00004-s001.zip › biology-1491041-supplementary.pdf]

Supplementary Information for

# Microcrater-arrayed Chemiluminescence Cell Chip to Boost Anti-cancer Drug Administration in Zebrafish Tumor Xenograft Model

Ching-Te Kuo[1*], Yu-Sheng Lai[2], Siang-Rong Lu[2,3], Hsinyu Lee[2] and Hsiu-Hao Chang[3**]

*[1]Department of Mechanical and Electro-Mechanical Engineering, National Sun Yat-sen University, Kaohsiung, Taiwan, R.O.C.*

*[2]Department of Life Science, National Taiwan University, Taipei, Taiwan, R.O.C.*

*[3]Department of Pediatrics, National Taiwan University Hospital and National Taiwan University College of Medicine, Taipei, Taiwan, R.O.C.*

*[*]Prof. Ching-Te Kuo. E-mail: chingtekuo@mail.nsysu.edu.tw or*
*[**]Dr. Hsiu-Hao Chang. E-mail: changhh2001@ntu.edu.tw*

Supplementary information includes 3 figures.

Supplementary Figures

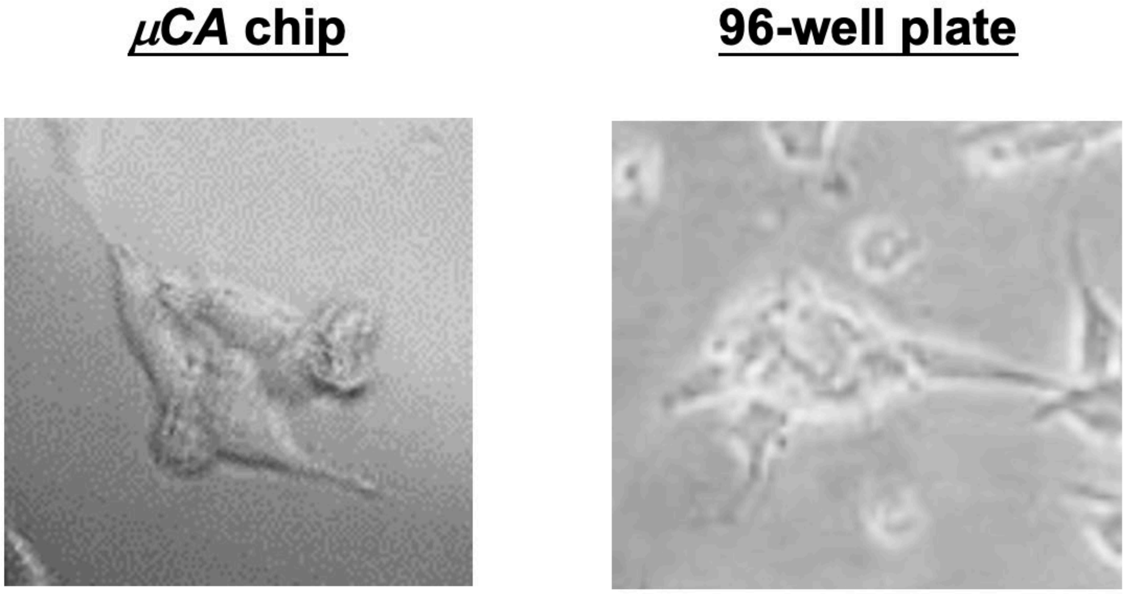


**Figure S1.** Cell morphology derived from the μCA chip and 96-well plate. The cells were SK-N-DZ cell lines.

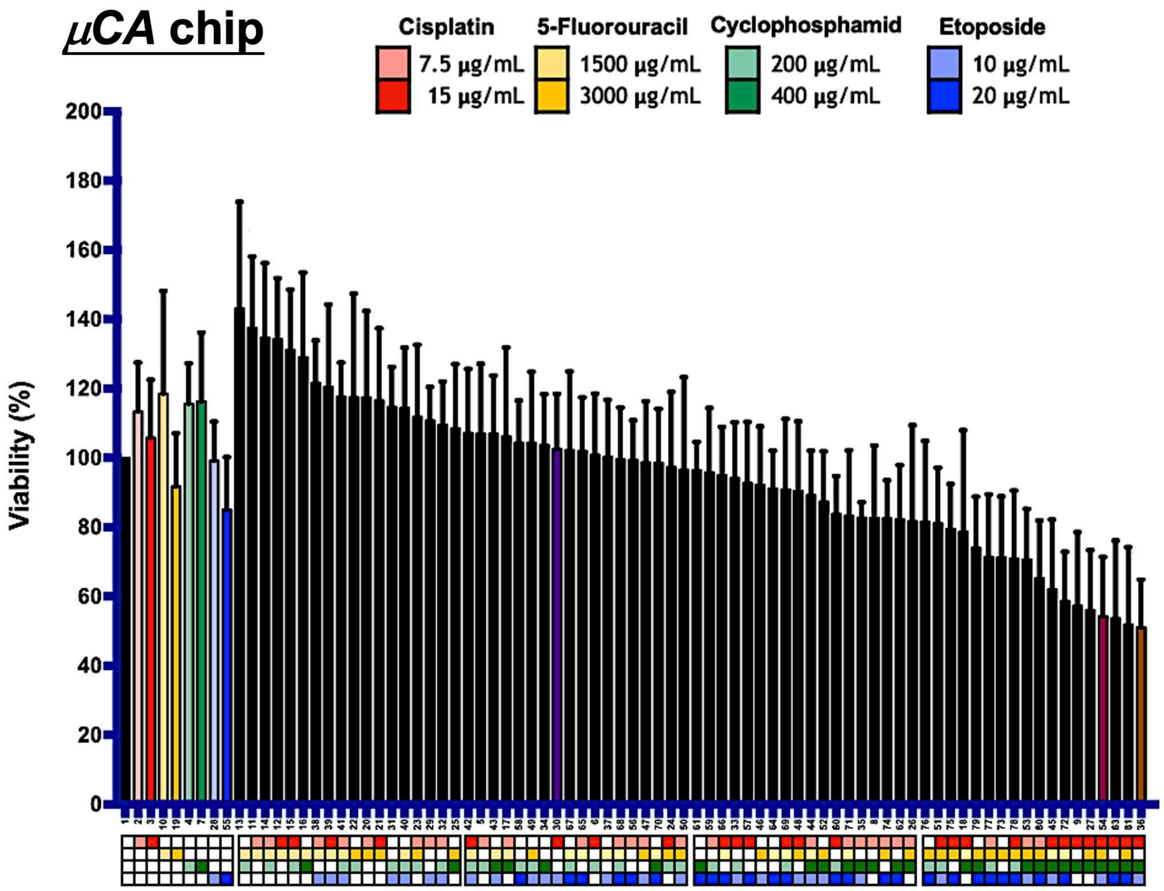


**Figure S2.** Cell viability of MCF-7 cells under the cocktailed treatments by the *μCA* chip. Drugs are selected to be cisplatin, 5-Fluorouracil, cyclophosphamide and etoposide with different concentrations. The four drug combinations selected for analysis are highlighted by purple (#30), dark red (#54) and brown (#36) colors, respectively. Data represent the mean ± SD, n = 3 independent experiments.

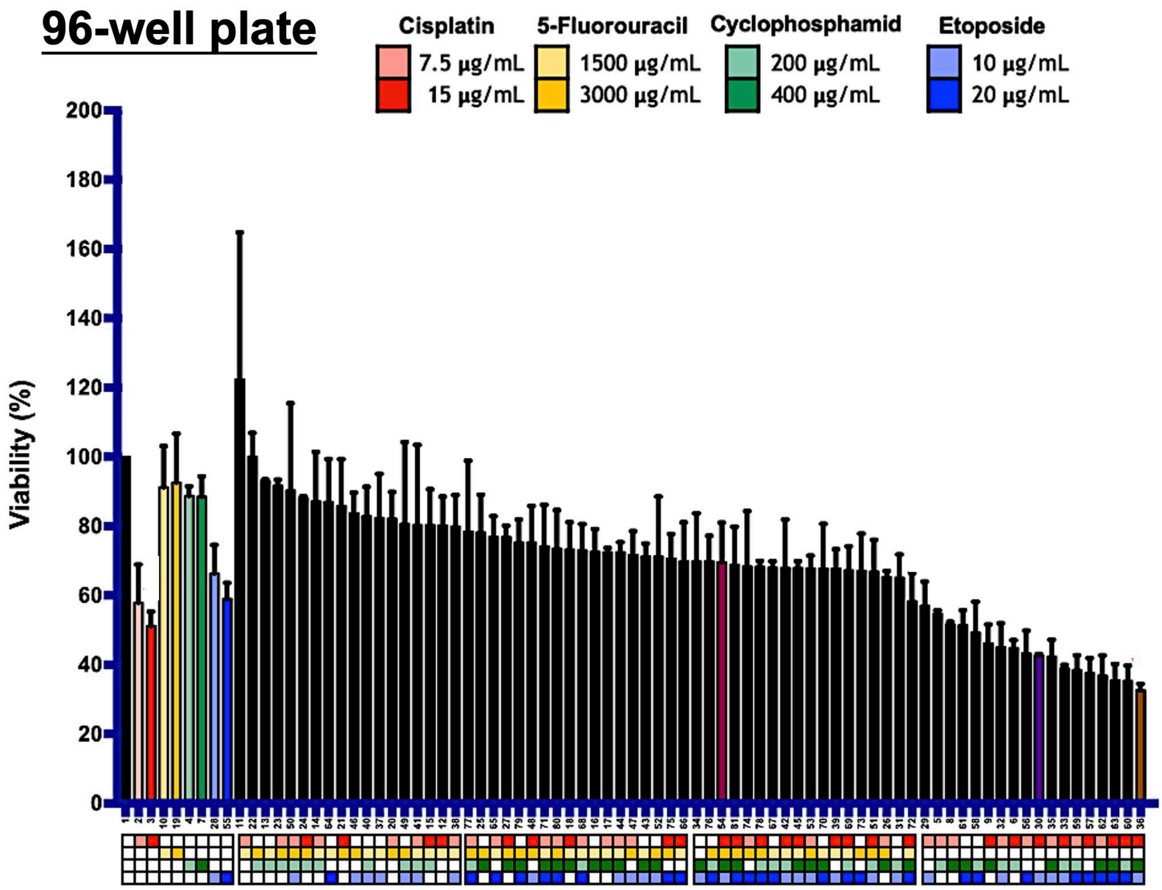


**Figure S3.** Cell viability of MCF-7 cells under the cocktailed treatments by the 96-well plate. Drugs are selected to be cisplatin, 5-Fluorouracil, cyclophosphamide and etoposide with different concentrations. The four drug combinations selected for analysis are highlighted by dark red (#54), purple (#30) and brown (#36) colors, respectively. Data represent the mean ± SD, n = 3 independent experiments.
